# Impact of ERAS on morbidity and length of stay in elderly patients undergoing elective laparoscopic colorectal cancer surgery: a two-center comparative study

**DOI:** 10.1007/s00423-026-04120-4

**Published:** 2026-06-25

**Authors:** Alessandro Verbo, Iacopo Verbo

**Affiliations:** 1 Department of General Surgery, Catholic University of the Sacred Heart, Rome, Italy; 2 Department of General Surgery, Sapienza University of Rome, Rome, Italy

**Keywords:** Enhanced recovery after surgery (ERAS), Colorectal cancer, Laparoscopic surgery, Postoperative complications, Length of stay, Elderly patients

## Abstract

**Background:**

Enhanced Recovery After Surgery (ERAS) protocols are widely implemented in colorectal surgery to improve postoperative recovery and reduce hospital stay. However, their impact on postoperative morbidity in elderly and clinically complex populations remains debated.

**Aim:**

To evaluate the impact of ERAS implementation on postoperative morbidity and hospital length of stay in an elderly and oncologically complex population undergoing elective laparoscopic colorectal cancer surgery.

**Methods:**

We conducted a retrospective comparative study including patients undergoing elective laparoscopic colorectal cancer resection. Patients treated between January 2016 and December 2018 received standard perioperative care, whereas those treated between January 2022 and December 2025 were managed according to a structured ERAS protocol. All procedures were performed laparoscopically by the same surgical team. To reduce selection bias and improve comparability between cohorts, propensity score matching was performed using age, sex, tumour location, T stage, N stage, and preoperative metastatic disease. The primary endpoint was the incidence of major postoperative complications (Clavien–Dindo grade ≥ III). Secondary endpoints included overall postoperative complications, in-hospital/30-day mortality, and length of hospital stay.

**Results:**

A total of 209 patients were included (ERAS: 83; non-ERAS: 126). Propensity score matching generated 68 matched pairs, achieving adequate covariate balance (all standardized mean differences < 0.10). In the overall cohort, major complication rates were similar between groups (22.9% vs. 23.8%, *p* = 1.000), as were overall complication rates (59.0% vs. 61.1%, *p* = 0.876). Mortality did not differ significantly (6.0% vs. 2.4%, *p* = 0.270). Length of hospital stay was significantly shorter in the ERAS group (6.2 ± 2.8 vs. 7.9 ± 5.7 days, *p* = 0.004).

**Conclusions:**

In an elderly and clinically complex population undergoing elective laparoscopic colorectal cancer surgery, ERAS implementation was associated with a significant reduction in hospital length of stay without evidence of increased major postoperative morbidity or mortality. Propensity score matching improved baseline comparability between cohorts. Given the retrospective design and non-contemporaneous study periods, these findings should be interpreted with caution but support the feasibility and safety of ERAS in real-world colorectal surgical practice.

## Introduction

Colorectal cancer remains one of the most common malignancies worldwide, and surgical resection continues to represent the cornerstone of curative treatment. Despite the widespread adoption of minimally invasive techniques, postoperative morbidity remains a major determinant of short-term outcomes, healthcare utilization, and recovery after surgery.

Enhanced Recovery After Surgery (ERAS) protocols were developed to optimize perioperative care through a multimodal, evidence-based approach aimed at reducing surgical stress, accelerating functional recovery, and shortening hospital stay. Current ERAS Society guidelines strongly recommend structured perioperative pathways in elective colorectal surgery. Multiple randomized trials and meta-analyses have consistently demonstrated reductions in hospital length of stay following ERAS implementation, whereas their impact on postoperative morbidity has been less consistent across studies.

In contemporary surgical practice, elderly patients and those presenting with advanced oncological disease represent an increasing proportion of the colorectal surgical population. These patients frequently exhibit greater physiological vulnerability and are often underrepresented in randomized clinical trials. Consequently, the effectiveness and safety of ERAS pathways in real-world elderly and clinically complex populations remain subjects of ongoing investigation.

Although several studies have reported favourable outcomes associated with ERAS implementation, interpretation of retrospective comparative studies may be limited by baseline differences between treatment groups and by potential selection bias. Statistical approaches such as propensity score matching have been increasingly adopted to improve comparability between cohorts and strengthen causal inference in observational surgical research.

The aim of the present study was to evaluate the impact of ERAS implementation on postoperative morbidity and hospital length of stay in an elderly and oncologically complex population undergoing elective laparoscopic colorectal cancer surgery. To reduce selection bias and improve comparability between cohorts, a propensity score matching analysis was performed in addition to conventional comparative analyses.

## Materials and methods

### Study design and patient population

This retrospective comparative study included consecutive patients undergoing elective laparoscopic colorectal cancer resection in two surgical centres. Patients treated in Hospital 1 between January 2016 and December 2018 received standard perioperative care, whereas patients treated in Hospital 2 between January 2022 and December 2025 were managed according to a structured Enhanced Recovery After Surgery (ERAS) protocol.

Only patients undergoing elective laparoscopic procedures for histologically confirmed colorectal cancer were included in the analysis. Emergency procedures and open surgeries were excluded to ensure a homogeneous cohort and minimize variability related to surgical urgency or operative approach.

All procedures in both centres were performed by the same surgical team according to standardized oncological principles. This approach minimized variability related to surgeon-dependent factors and technical evolution over time.

### ERAS protocol

The ERAS pathway implemented in Hospital 2 followed the principles outlined by the ERAS Society and included structured preoperative counselling, minimization of preoperative fasting, multimodal opioid-sparing analgesia, standardized perioperative fluid management, early mobilization, and early resumption of oral intake. The protocol also included avoidance of routine nasogastric decompression, early removal of urinary catheters whenever feasible, and encouragement of postoperative functional recovery according to ERAS principles. Implementation was protocol-driven, standardized, and consistently applied throughout the study period, ensuring uniform perioperative management within the ERAS centre.

### Data collection

Demographic, clinical, and pathological data were collected from institutional databases. Variables included age, sex, tumour location, pathological T stage, nodal status, presence of preoperative metastatic disease, postoperative complications, and length of hospital stay.

Postoperative complications were graded according to the Clavien–Dindo classification. The primary endpoint of the study was the incidence of major postoperative complications, defined as Clavien–Dindo grade ≥ III. Secondary endpoints included overall postoperative complications, in-hospital or 30-day mortality, and length of hospital stay.

### Propensity score matching

To reduce selection bias and improve comparability between cohorts, propensity score matching (PSM) was performed. Propensity scores were estimated using a logistic regression model including age, sex, tumour location, pathological T stage, nodal status, and preoperative metastatic disease. Patients were matched in a 1:1 ratio using nearest-neighbor matching without replacement. Covariate balance before and after matching was assessed using standardized mean differences (SMDs), with absolute values < 0.10 considered indicative of adequate balance.

### Statistical analysis

Continuous variables are presented as mean and standard deviation and were compared using Student’s t-test. Categorical variables are expressed as number and percentage and were compared using the chi-square test or Fisher’s exact test when appropriate. A p value of less than 0.05 was considered statistically significant.

### Ethical considerations

This study was conducted in accordance with the principles of the Declaration of Helsinki. Given the retrospective nature of the study and the use of anonymized data, formal approval by the institutional ethics committee was waived according to local regulations.

## Results

### Patient characteristics

A total of 209 patients undergoing elective laparoscopic colorectal cancer resection were included in the analysis: 83 in the ERAS group (Hospital 2) and 126 in the non-ERAS group (Hospital 1).

Before matching, nodal involvement was more frequent in the non-ERAS cohort. Propensity score matching was therefore performed using age, sex, tumour location, pathological T stage, nodal status, and preoperative metastatic disease to improve baseline comparability between groups.

Propensity score matching generated 68 matched pairs. After matching, all baseline covariates were adequately balanced, with absolute standardized mean differences below 0.10 (Table 1).

**Table 1:** Baseline characteristics before and after propensity score matching

Variable	Before matching ERAS (*n*=83)	Before matching Non-ERAS (*n*=126)	SMD before	After matching ERAS (*n*=68)	After matching Non-ERAS (*n*=68)	SMD after
Age (years), mean ± SD	71.0 ± 11.6	71.5 ± 10.8	0.045	71.9 ± 10.9	72.0 ± 11.1	0.008
Male sex, n (%)	56 (67.5)	76 (60.3)	0.149	47 (69.1)	47 (69.1)	0
Age ≥75 years, n (%)	36 (43.4)	54 (42.9)	0.01	32 (47.1)	32 (47.1)	0
Age ≥80 years, n (%)	22 (26.5)	33 (26.2)	0.007	18 (26.5)	18 (26.5)	0
Rectal tumor, n (%)	39 (47.0)	45 (35.7)	0.23	27 (39.7)	27 (39.7)	0
T0–T2 disease, n (%)	37 (44.6)	38 (30.2)	0.301	27 (39.7)	24 (35.3)	0.091
T3–T4 disease, n (%)	46 (55.4)	88 (69.8)	0.301	41 (60.3)	44 (64.7)	0.091
N+ disease, n (%)	29 (34.9)	62 (49.2)	0.292	23 (33.8)	20 (29.4)	0.095
M1 disease, n (%)	2 (2.4)	5 (4.0)	0.089	2 (2.9)	2 (2.9)	0

Data are presented as mean ± standard deviation (SD) or n (%)

Covariate balance was assessed using standardized mean differences (SMDs)

Absolute SMD values < 0.10 were considered indicative of adequate covariate balance

Propensity score matching was performed using 1:1 nearest-neighbor matching without replacement

### Postoperative outcomes

Postoperative outcomes in the overall study cohort are summarized in Table 2.

**Table 2:** Postoperative outcomes in the overall study cohort

Outcome	ERAS (*n*=83)	Non-ERAS (*n*=126)	*p* value
Any complication (Clavien–Dindo grade ≥ I), n (%)	49 (59.0)	77 (61.1)	0.876
Major complication (Clavien–Dindo grade ≥ III), n (%)	19 (22.9)	30 (23.8)	1,000
Mortality (Clavien–Dindo V), n (%)	5 (6.0)	3 (2.4)	0.270
Length of stay (days), mean ± SD	6.2 ± 2.8	7.9 ± 5.7	0.004

Data are presented as mean ± standard deviation (SD) or n (%)

Continuous variables were compared using Student’s t-test

Categorical variables were compared using the chi-square test or Fisher’s exact test, as appropriate

A *p* value < 0.05 was considered statistically significant

The incidence of major postoperative complications (Clavien–Dindo grade ≥ III), defined as the primary endpoint, was comparable between groups (22.9% in the ERAS group vs. 23.8% in the non-ERAS group, *p* = 1.000). Overall complication rates were also similar (59.0% vs. 61.1%, *p* = 0.876).

In-hospital or 30-day mortality did not differ significantly between groups (6.0% in the ERAS group vs. 2.4% in the non-ERAS group, *p* = 0.270).

Length of hospital stay was significantly shorter in the ERAS group compared with the non-ERAS group (6.2 ± 2.8 vs. 7.9 ± 5.7 days, *p* = 0.004).

## Discussion

The present study evaluated the impact of ERAS implementation in an elderly and oncologically complex population undergoing elective laparoscopic colorectal cancer surgery. The principal finding of this study is that ERAS was associated with a significant reduction in hospital length of stay without increasing major postoperative morbidity or mortality.

To improve comparability between cohorts and reduce selection bias, propensity score matching was performed using baseline demographic and oncological variables. Matching generated 68 well-balanced pairs, with all post-matching standardized mean differences below 0.10. These findings support the robustness of the comparative analysis despite the retrospective nature of the study.

The overall complication rate observed in our cohort, approximately 60%, reflects the characteristics of the study population. The mean age was 71 years, with 43% of patients aged ≥ 75 years and 26% aged ≥ 80 years. In addition, a substantial proportion of patients presented with advanced-stage disease. These features are consistent with contemporary real-world colorectal surgical populations and may explain the relatively high incidence of postoperative events. When complications are recorded using the Clavien–Dindo classification, including minor deviations from the expected postoperative course, overall morbidity rates between 40% and 60% are frequently reported in large series [1, 2].

Importantly, the incidence of major complications in our study (approximately 23%) falls within the upper range described in high-volume colorectal centres and registry-based analyses [3–5]. Propensity score matching achieved adequate balance between groups, reducing the potential influence of baseline differences on postoperative outcomes.

ERAS implementation did not result in a reduction in major or overall complication rates. This observation is consistent with previous evidence. While randomized trials and meta-analyses have consistently demonstrated reduced hospital stay following ERAS adoption, their impact on major postoperative morbidity has been less uniform [6–9]. The ERAS Compliance Group has similarly shown that protocol adherence improves recovery metrics and organizational outcomes, although reductions in severe morbidity are not consistently observed [3]. Our results therefore align with existing literature and reinforce the concept that ERAS primarily optimizes perioperative recovery rather than fundamentally altering surgical risk.

The most robust finding of our study is the significant reduction in hospital length of stay in the ERAS group (6.2 ± 2.8 vs. 7.9 ± 5.7 days). This reduction was achieved without any increase in major complications or mortality, supporting the safety of ERAS implementation even in elderly and potentially fragile patients. The absence of increased morbidity suggests that protocol-driven perioperative optimization does not compromise surgical safety in high-risk cohorts.

Several limitations should be acknowledged. First, the retrospective design exposes the study to potential selection bias and unmeasured confounding. Although propensity score matching improved comparability between cohorts, residual confounding due to variables not available in the database cannot be completely excluded. Second, the two cohorts were derived from different time periods, and improvements in perioperative care, anaesthetic management, and institutional experience may have influenced outcomes independently of ERAS implementation. Nevertheless, all procedures were performed laparoscopically by the same surgical team according to standardized oncological principles, partially mitigating variability related to surgeon experience or technical evolution. Third, detailed comorbidity measures such as ASA score, Charlson Comorbidity Index, frailty assessment, BMI, and nutritional parameters were not consistently available across both historical cohorts and therefore could not be incorporated into the propensity score model. Finally, compliance with individual ERAS components was not prospectively recorded and could therefore not be analysed, preventing assessment of the relationship between protocol adherence and clinical outcomes. In addition, data regarding unplanned readmissions and unscheduled postoperative consultations were not consistently recorded in the institutional databases and therefore could not be reliably analysed.

## Conclusion

In an elderly and oncologically complex population undergoing elective laparoscopic colorectal cancer surgery, ERAS implementation was associated with a significant reduction in hospital length of stay without evidence of increased major postoperative morbidity or mortality. Propensity score matching improved baseline comparability between cohorts and reduced the potential impact of selection bias. Although residual confounding inherent to retrospective studies cannot be completely excluded, these findings support the safety and organizational benefits of ERAS in real-world colorectal surgical practice. 

## Data Availability

The datasets generated and analyzed during the current study are available from the corresponding author upon reasonable request.
